# Computational Screening to Predict MicroRNA Targets in the Flavivirus 3′ UTR Genome: An Approach for Antiviral Development

**DOI:** 10.3390/ijms251810135

**Published:** 2024-09-21

**Authors:** Rodolfo Gamaliel Avila-Bonilla, Juan Santiago Salas-Benito

**Affiliations:** 1 Laboratorio de Genómica y Biología Molecular de ARNs, Departamento de Genética y Biología Molecular, Cinvestav, Av. IPN 2508, Mexico City 07360, Mexico; 2 Laboratorio de Biomedicina Molecular 3, Escuela Nacional de Medicina y Homeopatía, Instituto Politécnico Nacional, Mexico City 07320, Mexico

**Keywords:** flaviviruses, microRNA, 3′ UTR, computational analysis

## Abstract

MicroRNAs (miRNAs) are molecules that influence messenger RNA (mRNA) expression levels by binding to the 3′ untranslated region (3′ UTR) of target genes. Host miRNAs can influence flavivirus replication, either by inducing changes in the host transcriptome or by directly binding to viral genomes. The 3′ UTR of the flavivirus genome is a conserved region crucial for viral replication. Cells might exploit this well-preserved region by generating miRNAs that interact with it, ultimately impacting viral replication. Despite significant efforts to identify miRNAs capable of arresting viral replication, the potential of all these miRNAs to interact with the flavivirus 3′ UTR is still poorly characterised. In this context, bioinformatic tools have been proposed as a fundamental part of accelerating the discovery of interactions between miRNAs and the 3′ UTR of viral genomes. In this study, we performed a computational analysis to reveal potential miRNAs from human and mosquito species that bind to the 3′ UTR of flaviviruses. In humans, miR-6842 and miR-661 were found, while in mosquitoes, miR-9-C, miR-2945-5p, miR-11924, miR-282-5p, and miR-79 were identified. These findings open new avenues for studying these miRNAs as antivirals against flavivirus infections.

## 1. Introduction

Flaviviruses are characterised by a single-stranded RNA genome of approximately 11 kb with positive polarity [1]. Some of these, called mosquito-borne flaviviruses (MBFV), include pathogens such as dengue virus (DENV), Zika virus (ZIKV), yellow fever virus (YFV), West Nile virus (WNV), Japanese encephalitis virus (JEV), Saint Louis encephalitis virus (SLEV), Usutu virus (USUV), and Murray Valley encephalitis virus (MVEV) [2]. The viral genome consists of a single open reading frame (ORF) flanked by 5′ and 3′ untranslated regions (UTRs) [3]. The 3′ UTR is highly conserved across flaviviruses and comprises an initial variable region (VR), a central core, and terminal 3′-end regions [4,5]. This region plays a critical role in viral translation, replication, adaptation, fitness, virulence, and tissue tropism [6,7].

MicroRNAs (miRNAs) are a class of noncoding RNAs (ncRNAs), approximately 22 nucleotides (nt) in length, that are derived from longer primary miRNA (pri-miRNA) transcripts or processed by endogenous introns from snoRNAs, tRNAs, and shRNAs bearing one or more hairpins [8]. They are processed by two cellular RNase III enzymes, Drosha and Dicer, to generate mature miRNAs capable of controlling gene expression at the post-transcriptional level [9,10,11]. Mature miRNAs can be loaded onto Argonaute (AGO) proteins, allowing gene repression. Interestingly, miRNA target sites are typically located in the 3′ UTRs of mRNAs with strong complementarity to the seed region, which is the principal criterion for target-site prediction [12,13,14]. The binding of AGO–miRNA to the 3′ UTR of mRNAs leads to gene silencing by causing translational repression and promoting mRNA decay [15,16,17].

Indeed, miRNAs can interact with the 3′ UTR of the viral genome, exerting a significant influence on the viral replication cycle [18]. For instance, miR-484, miR-744 [19], and miR-133a [20] possess specific target sequences within the 3′ UTR of dengue virus (DENV) serotypes, and their overexpression inhibits viral replication in mammalian cell lines. Moreover, experimental results indicate that the introduction of miRNA recognition elements (MREs) into the 3′ UTRs of genetically modified flaviviruses has important implications for viral attenuation [21]. For instance, the incorporation of MREs for miR-122 [22] and miR-142 [23] into genetically modified dengue virus vaccine candidates increases the susceptibility of the virus to infection inhibition in cell models that overexpress these miRNAs. In addition, the insertion of miR-124 MRE into the JEV genome results in the inhibition of its replication and translation. This modified virus exhibits an attenuated phenotype in mice inoculated either intraperitoneally or intracerebrally and replicates inefficiently in the brain, where miR-124 is highly expressed, but shows no significant impact in the spleen or liver [24].

Furthermore, exploring interactions between flavivirus 3′ UTRs and host miRNAs provides promising avenues for pioneering strategies that harness the potential of these small RNAs to modulate viral replication [18,25,26,27,28]. Recently, 30 human microRNAs capable of recognising the 3′ UTRs of all four serotypes of DENV were reported [29]. However, it remains unknown whether these endogenous miRNAs can also recognise the 3′ UTRs of different flaviviruses or recognise the 3′ UTR in the transmission vector.

This ongoing study employs computational analyses to scrutinise the interactions between diverse human and mosquito host microRNAs and the 3′ UTRs of various members of the *Flaviviridae* family with medical importance. The goal is this study is to develop a fast and reliable approach for identifying new miRNAs in human and mosquito cells with therapeutic potential for regulating viral replication by interacting with the 3′ UTRs of several flaviviruses.

## 2. Results

### 2.1. Data Filtering of miRNA–Flavivirus Interactions

**Figure 1:**
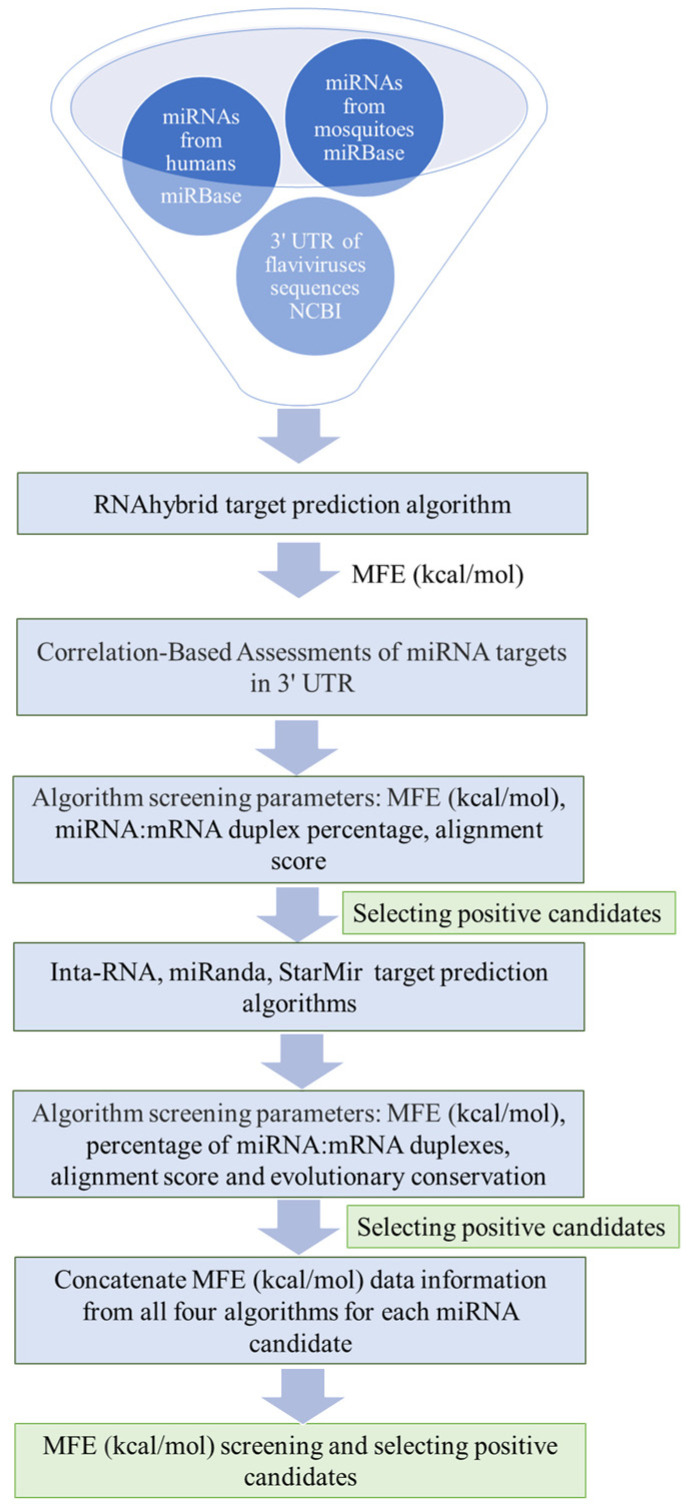
Workflow diagram for identifying new miRNAs in human and mosquito cells that can interact with the 3′ UTR of flaviviruses.

A dataset of 2693 mature human miRNAs, 165 from *Aedes (Ae.) aegypti* and 93 from *Culex (Cu.) quinquefasciatus*, was obtained from miRBase. Interactions between these miRNAs and flavivirus 3′ UTRs were assessed using RNAhybrid. Interactions with scores below −20 kcal/mol, utilising MFE as a stability metric, were considered. As a negative control, a dataset from *Caenorhabditis elegans*, which is not a natural host for these viruses, was incorporated. The results from this control were treated as algorithmic noise, and these miRNAs were excluded from further analyses. Supplementary Table S1 presents the results of the miRNAs identified by the RNAhybrid algorithm. An overview of our proposed methodology is illustrated in Figure 1. Remarkably, 29.40% of human miRNAs (792 miRNAs) exhibited interactions with at least one of the eleven flavivirus 3′ UTRs. In mosquitoes, the percentage was notably higher. For instance, of the total miRNAs present in *Ae. aegypti* and *Cu. quinquefasciatus*, 89.69% (148 miRNAs) and 73.34% (71 miRNAs), respectively, target at least one flavivirus 3′ UTR.

### 2.2. Human miRNA Interactions with the Flavivirus 3′ UTRs

The RNAhybrid MFE (kcal/mol) data were used to perform correlation analyses using Spearman’s correlation coefficient to evaluate which 3′ UTRs among the diverse flaviviruses exhibited stronger affinities for host miRNAs. Correlation values close to 1 indicate a strong positive association, suggesting that consistently lower MFE values, which reflect stronger binding, are observed among certain flaviviruses. This implies the presence of shared miRNA binding sites across these viruses, highlighting consistent patterns of interaction strength across different flaviviruses.

**Figure 2:**
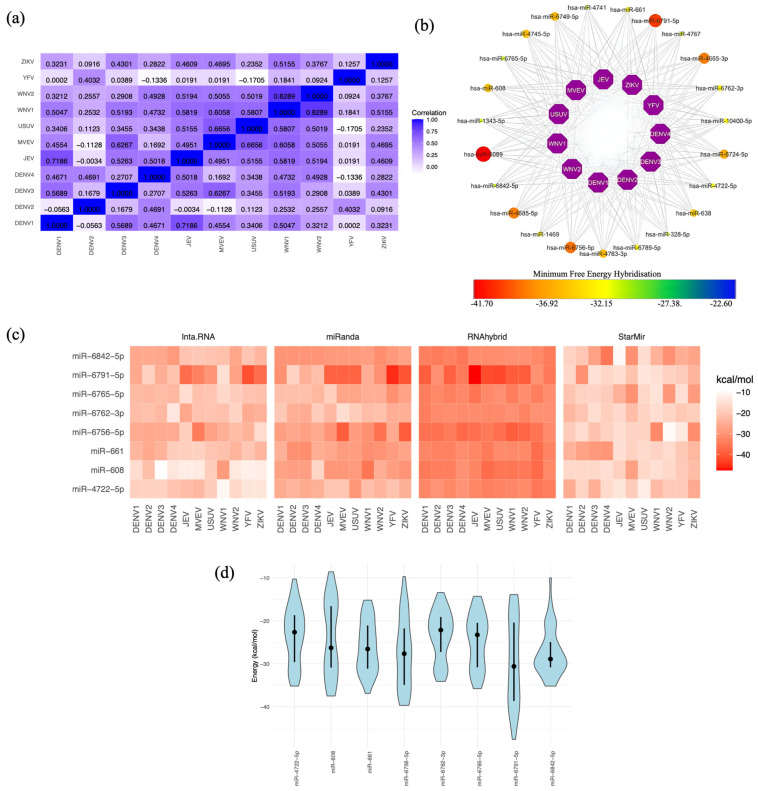
Human miRNA targets in flavivirus 3′ UTRs. (**a**) Spearman’s correlation coefficient analysis of the MFE for miRNAs targeting at least one 3′ UTR of the eleven flaviviruses. MFE values were calculated using the RNAhybrid algorithm, revealing values lower than −20 kcal/mol. (**b**) Network of miRNA candidates identified by RNAhybrid as targeting the genomes of all flaviviruses, with MFE values represented on a gradient from lower values in yellow dots to higher values in red dots. The miRNAs represented by the largest red dots correspond to those with the highest MFE scores, indicating potentially stronger binding interactions. (**c**) miRNA candidates selected from the results of the four algorithms. MFE values are represented in kcal/mol, with lower values shown in intense red. (**d**) Violin plot depicting the distribution of MFE values for miRNA candidates, featuring the mean (represented by black dots) and quartiles (Q3 and Q4) as intersecting lines. The MFE distribution was calculated using data from the four algorithms. The width of each violin represents the density of data points at different MFE values, with wider sections indicating a higher concentration of values in that range.

Notable correlations were evident in human miRNA–3′ UTR interactions (Figure 2a), such as the significant correlation between DENV1 and DENV3 (0.56), and the strong correlation between JEV and DENV1 (0.71). Additionally, DENV3 exhibited a strong correlation with ZIKV (0.43), while YFV displayed positive correlations with DENV2 (0.40) and WNV1 (0.18), indicating their active involvement in these interactions. MVEV exhibited a correlation with DENV1 (0.45) and WNV2 (0.50). Remarkably, WNV1 displayed a strong correlation with JEV (0.58). In addition to being positively correlated with DENV3, ZIKV was also positively correlated with JEV, MVEV (0.46), and WNV1 (0.51). All these correlations suggest possible similarities in miRNAs with affinity for the 3′ UTRs of the flavivirus genome.

### 2.3. Selecting the Optimal Human miRNA That Targets Flavivirus 3′ UTRs

We further evaluated 23 human miRNAs that exhibited binding to the 3′ UTRs of all eleven flaviviruses (Figure 2b). However, our analysis with three additional programs unexpectedly revealed that only eight human miRNAs consistently interacted with all eleven 3′ UTR sequences (Figure 2c). The target positions and corresponding 3′ UTR target sequences for these eight candidates are presented in Table 1. The distinct methodologies of each algorithm to determine the MFE in the miRNA–3′ UTR interactions allowed us to explore the MFE distribution for these eight miRNAs (Figure 2d). As anticipated, due to algorithmic differences, the MFE data showed variability. Notably, miR-6842-5p and miR-661 exhibited a greater degree of similarity in MFE predictions. This discovery emphasises the potential importance of miR-6842-5p and miR-661 in targeting the flavivirus 3′ UTR, making them compelling candidates for further research and potential therapeutic applications.

### 2.4. Mosquito miRNA Interactions with Flavivirus 3′ UTRs 

**Figure 3:**
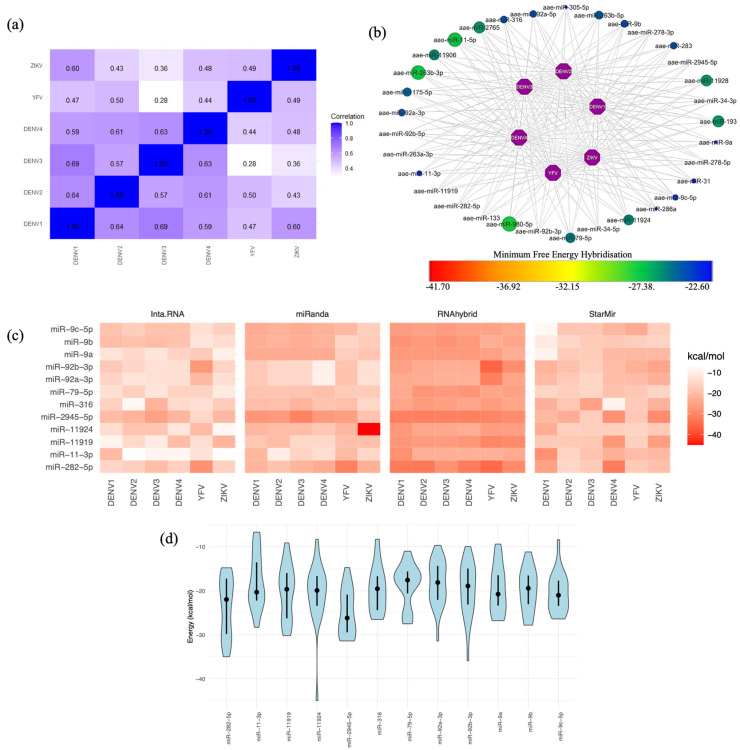
*Ae. aegypti* miRNA targets in flavivirus 3′ UTRs. (**a**) Spearman’s correlation coefficient analysis of the MFE for miRNAs targeting at least one 3′ UTR of the six flaviviruses. MFE values were calculated using the RNAhybrid algorithm, revealing values lower than −20 kcal/mol. (**b**) Network of miRNA candidates identified by RNAhybrid as targeting the genomes of all flaviviruses, with MFE values represented on a gradient from lower values in blue dots to higher values in green dots. The miRNAs represented by the largest green dots correspond to those with the highest MFE scores, indicating potentially stronger binding interactions. (**c**) miRNA candidates selected from the results of the four algorithms. MFE values are represented in kcal/mol, with lower values shown in intense red. (**d**) Violin plot depicting the distribution of MFE values for miRNA candidates, featuring the mean (represented by black dots) and quartiles (Q3 and Q4) as intersecting lines. The MFE distribution was calculated using the results from all four algorithms. The width of each violin represents the density of data points at different MFE values, with wider sections indicating a higher concentration of values in that range.

**Figure 4:**
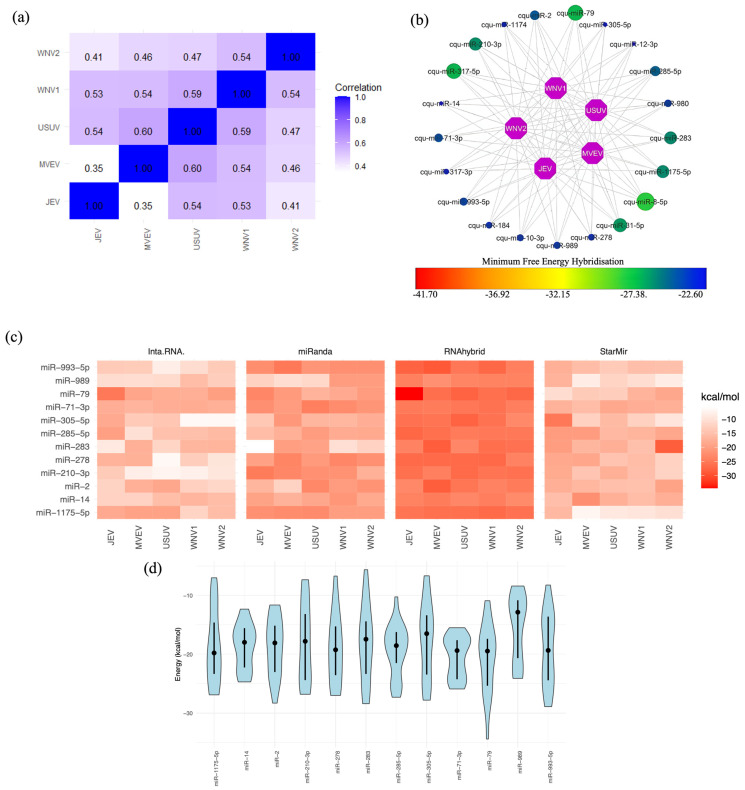
*Cu. quinquefasciatus* miRNA targets in flavivirus 3′ UTRs. (**a**) Spearman’s correlation coefficient analysis of MFEs for miRNAs targeting at least one 3′ UTR of the five flaviviruses. MFE values were calculated using the RNAhybrid algorithm, revealing values lower than −20 kcal/mol. (**b**) Network of miRNA candidates identified by RNAhybrid as targeting the genomes of all flaviviruses, with MFE values represented on a gradient from lower values in blue dots to higher values in green dots. The miRNAs represented by the largest green dots correspond to those with the highest MFE scores, indicating potentially stronger binding interactions. (**c**) miRNA candidates selected from the results of the four algorithms. MFE values are represented in kcal/mol, with lower values shown in intense red. (**d**) Violin plot depicting the distribution of MFE values for miRNA candidates, featuring the mean (represented by black dots) and quartiles (Q3 and Q4) as intersecting lines. The MFE distribution was calculated using the results from all four algorithms. The width of each violin represents the density of data points at different MFE values, with wider sections indicating a higher concentration of values in that range.

In our analysis of mosquito miRNAs and their interactions with flaviviruses, we segregated the data based on the mosquito species (Figure 3 and Figure 4). Unexpectedly, positive correlations in MFE values were observed for most viruses in both mosquito species, as shown in Figure 3a and Figure 4a. This indicates a strong affinity between mosquito miRNAs and the 3′ UTRs of these viruses. Several of these correlations were detected in both vertebrate and invertebrate organisms. For example, the correlation of DENV1 with DENV3 and DENV4 in humans and *Ae. aegypti* mosquitoes showed the highest correlation coefficient with DENV3 (0.56 vs. 0.59; compare Figure 2a with Figure 3a). DENV2 showed positive and significant correlation with DENV4 and YFV in both species, with the highest correlation being with DENV4 (0.46 vs. 0.61; compare Figure 2a with Figure 3a). A stronger correlation was observed between miRNAs in humans and *Cu. quinquefasciatus* mosquitoes (compare Figure 2a with Figure 4a). For example, WNV1 and 2 are strongly correlated with JEV, MVEV, and USUV in both organisms.

### 2.5. Selecting the Optimal Mosquito miRNA That Targets Flavivirus 3′ UTRs 

In *Ae. aegypti*, we identified 33 miRNAs that target the 3′ UTRs of six flaviviruses (Figure 3b). In *Cu. quinquefasciatus*, 21 miRNAs interacted with the 3′ UTRs of five flaviviruses (Figure 4b). In both cases, 12 miRNAs were predicted by the four programs to interact with the 3′ UTRs of flaviviruses (Figure 3c and Figure 4c). The target positions and corresponding 3′ UTR target sequences for these 12 miRNA candidates are presented in Table 2 for *Ae. aegypti* and Table 3 for *Cu. quinquefasciatus*. Finally, the distribution of MFEs for these miRNAs is displayed, revealing variability and, in some cases, MFE values exceeding −20 kcal/mol. This discrepancy might be attributable to the fact that the programs were not originally designed for invertebrate miRNAs. Nonetheless, the average MFEs for certain miRNAs remained below −20 kcal/mol, indicating their potential as promising candidates. Additionally, some miRNAs displayed consistent MFE predictions across different programs. Notable candidates among *Ae. aegypti* mosquito miRNAs include miR-9-C-5p, miR-2945-5p, miR-11924, and miR-282-5p (Figure 3d). In the case of *Cu. quinquefasciatus*, the standout candidate was miR-79 (Figure 4d).

## 3. Discussion

miRNAs are considered good therapeutic agents because they are small molecules, have an endogenous origin and flexible functions, do not induce a relevant immune response, do not have important side effects and, since their mechanism of action does not require full complementarity to the target sequence, they tolerate mutations outside the seed region [30,31,32]. Additionally, synthetic miRNAs, contrary to cellular RNAs, are more stable and resistant to degradation by environment deleterious conditions [30].

There are several studies about the participation of miRNAs in the flavivirus replicative cycle using human cell lines as a model. The most common process regulated by miRNAs during flavivirus infection is the innate immune response and inflammation and includes miR-146a-5p [33] in DENV infection; miR-146a in DENV [34] and ZIKV [35] infections; miR-532-5p in WNV infection [36]; and miR-19b-3p [37], miR-9-5p [38], and miR-15b [39] in JEV neuroinflammation. Finally, BACH1, a transcriptional repressor of HO-1 that participates in the regulation of the IFN response, is the target gene of miRNAs that are dysregulated in DENV infections, like let-7c [40] and miR-155 [41]. miR-155 also participates in the inflammatory process during JEV infection [42].

Human miRNAs also participate in other processes required for flavivirus infection, like miR-383-5p in lipid metabolism during DENV infection, which affects viral particle production [43]; miR-15 and -16, which increase the activity of caspases 3/7, indicating a probable relationship with apoptosis, also in DENV infection [44]; miR-3614-5p, which reduces DENV2 and WNV infection by inhibiting the action of adenosine deaminase on RNA 1 (ADAR1), a factor that promotes viral infectivity in early stages of infection [45]; miR-532-5p, which reduces the expression of TAB3, a factor involved in cell survival, proliferation, differentiation, embryonic development, inflammation, and carcinogenesis, and SESTD1, a phospholipid-binding protein essential for the efficient activation of the calcium channels TRPC4 and TRPC5, which is required for efficient propagation of WNV [36]; miR-33a-5p, which has an inhibitory effect on viral replication by silencing the EEF1A1 factor, a component of the JEV replication complex that avoids NS3 and NS5 proteasome degradation [46]; and, finally, miR-204-5p and miR-103a-3p, whose expression is induced by ZIKV E protein [47] and infection [48], respectively. miR-204-5p downregulates WNT2, a growth factor that is involved in brain development [47], and miR-103a-3p promotes the phosphorylation of p38 MAPK and HSP2 through the inhibition of OTUD4 [48].

The information about the participation of miRNAs in flavivirus infections in mosquitoes is more limited. Again, the innate immune response is regulated by miRNAs like miR-375 in *Ae. aegypti* mosquitoes infected with DENV2 [49]. miR-252 is downregulated in *Ae. albopictus* mosquitoes infected with DENV2, and this miRNA has a target sequence in the viral E protein, suggesting an antiviral role [50]. Finally, miRNA-240-5p is specifically downregulated in the *Ae. albopictus* cell line C6/36 when infected by WNV in a time-dependent manner. This miRNA is involved in the translation regulation of m41 FtsH, an ATP-dependent metalloprotease that localises to the inner membrane of mitochondria and that is responsible for the degradation of misfolded proteins. The silencing of this protein results in the reduction of both the viral titre and the quantity of viral genomes in infected cells, indicating its relevance in the WNV replicative cycle [51].

Since miRNAs play important roles in viral infections, they have been seriously considered for the treatment of several viral infections, and they have been tested in in vivo preclinical trials [52]. For example, the design of synthetic miRNAs against the 3′ and 5′ UTRs and ORF9 of Severe Acute Respiratory Syndrome Coronavirus 2 (SARS-CoV2) has been proposed to inhibit the translation process [30]. A similar approach has been used to reduce coxsackievirus B3 (CVB3) infection in HeLa cells. In this case, two artificial miRNAs (AmiR) against the Y loop of the viral 3′ UTR, delivered to HeLa cells by folate-mediated internalisation via the folate receptor, demonstrated their antiviral effect [32]. The transfection of miR-199a-3p and miR-210 into HepG2 2.2.15 cells, which target the S and P regions of the genome of Hepatitis B virus (HBV), reduced the expression of the S antigen (HBsAg) and viral replication [53].

Finally, miRNAs have been proposed for the design of viral vaccines. The insertion of an MRE into the ORF of the nucleoprotein of influenza virus A H1N1 and H5NI allowed the generation of an influenza vaccine that displays an attenuated phenotype in mice but not in eggs [54]. The insertion of MREs for miR-133 and miR-206 into the 5′ UTR of CVB3 resulted in the generation of engineered viruses that could replicate efficiently in HeLa but not in TE671 cells or mice cardiac muscle. Additionally, these viruses were able to induce neutralising anti-CVB3 antibodies and protect against wild-type virus challenge in mice [55]. Similar results were obtained with engineered viruses that included MREs specific for different tissues (miR-206, specific for muscle; miR-29a, specific for pancreas; and miR-124-3p, abundant in the central nervous system) [56].

Bioinformatic analysis is a time-efficient approach for approximating miRNA interactions with 3′ UTRs of the viral genome [18]. This computational approach has been successfully applied to various RNA viruses, including influenza C virus [57] and DENV [29]. Our research extends the analysis to various mosquito-borne flaviviruses, including both their vectors and human hosts. The most crucial step for gene or mRNA silencing is the effective hybridisation and heteroduplex formation between miRNAs and 3′ UTRs [58]. Seed types (8mer, 7mer-A1, and 7mer-m8) have been noted to be particularly recognisable by the RNA-induced silencing complex (RISC), increasing the degree of gene silencing [59,60,61].

The target prediction algorithms employed in this study are considered the most suitable for achieving the effective identification of miRNA binding sites on the 3′ UTRs of the flavivirus genome. The MFE and seed region of the miRNA–target hybrid are consistently recognised as the most widely exploited parameters in all these miRNA target prediction algorithms [62,63,64,65,66]; therefore, these parameters were selected to process the data with the utmost rigor to avoid false-positive candidates. The information obtained from the four algorithms was concatenated to identify the miRNAs with the highest scores and the potential for binding to the 3′ UTRs.

Among the identified interactions, the human miRNAs miR-6842 and miR-661 demonstrated robust targeting of flavivirus 3′ UTRs. Notably, miR-661, previously identified as a promising miRNA in interactions with all four DENV serotypes [29], retained its importance in our study. Additionally, miR-484, miR-744 [19], and miR-133a [20] have been reported to possess specific target sequences within the DENV 3′ UTR. While our initial analysis detected these miRNAs as targets of at least one flavivirus (see Supplementary Table S1), they did not exhibit interactions with all 11 proposed flaviviruses. This finding underscores the potential of other miRNAs with broader binding capabilities to exert effects against these viruses.

However, all these predictions should be validated by experimental approaches. For example, miR-532 has two putative binding sites in the WNV genome predicted by the RNAhybrid algorithm that are not functional in vitro [36]. For these validations, a common approach is to clone the putative target sequence in the 3′ UTR of a luciferase gene to perform a double-luciferase reporter gene assay [35,36,37,38,39,41,47,48]. Then, the antiviral effect of the miRNA should be evaluated during a flavivirus infection. For that, a miRNA mimic is transfected into a suitable cell line and then infected with a flavivirus. There are several suitable human cell lines that can be used for this purpose, like Huh-7 [33,40,41,43,44,45], THP1 [34], HepG2 [44], HEK293 [36,46], HCM3 [35,42], U251 [37,39], SH-SY5Y [38], and A549 cells [48]. The C6/36 cell line from *Ae albopictus* is a suitable model for performing these experiments with mosquito miRNAs [67]. The infection can be evaluated by determining viral titres by the plaque assay [36,39,45,46,67], the viral genome copy number by RT-qPCR [40,41,44,45,67], and viral protein synthesis by Western blotting [36,37,38,39,41,46,48].

However, our mosquito-focused analysis faced challenges, because most algorithms have been created for mammalian miRNA interactions, and mosquito miRNA processing differs substantially [68,69,70]. Despite these limitations, the positive MFE correlations observed for the interactions between mosquito miRNAs and the 3′ UTRs of flaviviruses are particularly intriguing, indicating a substantial affinity between mosquito miRNAs and the 3′ UTRs of various flaviviruses. In contrast, human interactions demonstrated negative correlations when evaluating different flaviviruses. These differences may have significant biological implications for mosquito vectors and warrant further experimental exploration. Additionally, we identified miRNAs that potentially interact with *Aedes* and *Culex* mosquitoes, including miR-9c, miR-2945-5p, miR-11924, and miR-282-5p, while miR-79 emerged as a noteworthy candidate in the context of flavivirus infections. Given that not all algorithms were created with insects in mind, it is important to highlight that future work can further develop this approach to define more precisely the interactions of mosquito miRNAs with the 3′ UTRs.

On the other hand, there are no experimental reports of the participation of these miRNAs in flavivirus infections, and only human miR-661 has been determined to be notably increased in the serum of patients with herpes zoster infection. Using TargetScan (Version 7.1) software, several target genes of this miRNA were identified; these were associated with the nervous and immune systems, but none of them were validated experimentally [71]. However, the functions of some of these miRNAs have been reported. For example, in invertebrates (see Table 4), miR-9c is involved in the development of the fruit fly *Drosophila melanogaster* [72,73,74] and the mud crab *Scylla paramamosain* [75,76]; miR-282-5p is involved in the moulting process of the silkworm *Bombyx mori* [77]; and finally, miR-79, the orthologue of miR-9 in humans [78], participates in cell proliferation and development in several organisms such as the fruit fly [74,79], worm [78], silkworm [80], and sea cucumber [81]. Interestingly, it has been reported that miR-79 is overexpressed in ISE6 cells from the tick I. scapularis infected with the bacterium *Anaplasma phagocytophilum*. This miRNA suppresses the expression of Roundabout protein 2 (Robo2), a molecule involved in the proinflammatory response, thereby promoting infection [82]. Additionally, miR-79 has been shown to be upregulated in exosomes from patients with chronic rhinosinusitis with nasal polyps [83].

More information is available for human miRNAs. For example, miR-6842-5p is involved in glucose metabolism through the inhibition of AKT2 and has a negative effect on proliferation and migration in endothelial cells during persistent high-glucose exposure, and miR-661, one of the most promising miRNAs identified in this work, has been implicated in several types of cancer as well as some diseases, such as diabetes mellitus 2 and Alzheimer’s disease (see Table 5). The present work identified new potential functions of these miRNAs through the proposed computational workflow. These miRNAs have the potential to be utilised as tools in the development of antiviral therapies, as both miRNAs have binding sites in the 3′ UTRs of flaviviruses [77]. These regions play crucial role in the post-transcriptional repression and decay of RNAs [15,16,17,84], so defining their functions within virus infections will have important implications for future therapeutic endeavours.

## 4. Materials and Methods

### 4.1. Retrieval of the Viral Genome and Mature miRNA Sequences

The genomic sequences of the flaviviruses DENV1 (accession NC_001477.1), DENV2 (accession NC_001474.2), DENV3 (accession NC_001475.2), DENV4 (accession NC_002640.1), WNV1 (accession NC_009942.1), WNV2 (accession NC_001563.2), ZIKV (accession NC_035889.1), YFV (accession NC_002031.1), JEV (accession NC_001437.1), MVEV (accession NC_000943.1), and USUV (accession NC_006551.1) were retrieved from the National Center for Biotechnology Information (NCBI) GenBank platform “https://www.ncbi.nlm.nih.gov/ (accesed on 27 August 2024)”. FASTA-formatted genomic sequences of flaviviruses were processed using bedtools “https://bedtools.readthedocs.io/en/latest/ (accesed on 27 August 2024)” to extract their 3′ UTRs.

miRNA sequences from humans (Homo sapiens; hsa.gff3); two mosquito species, *Aedes aegypti* (aae.gff3) and *Culex quinquefasciatus* (cfa.gff3); and *Caenorhabditis elegans* (cel.ggf3) were obtained from the miRBase database “https://www.mirbase.org (accesed on 27 August 2024).

### 4.2. miRNA Target Site Algorithms

To identify endogenous miRNA target sites in the 3′ UTR sequences of different flaviviruses, four reliable target prediction algorithms were employed in this study:

RNAhybrid: This algorithm calculates the minimum free energy (MFE) for miRNA–target hybrids using thermodynamic principles. It integrates helix parameters and loop constraints and accounts for G:U wobbles within the seed region. A favourable free energy for hybridisation is typically approximately −20 kcal/mol [43].
Inta-RNA: Using an enhanced scoring system, this algorithm predicts RNA–RNA interactions. It assesses the thermodynamic stability of interaction duplexes, site accessibility, and seed region attributes. Interactions are predicted when both the total energy and hybridisation energy are less than zero, with scores greater than 140 indicating optimal interactions [44].
miRanda: This algorithm identifies miRNA–mRNA target duplexes, accommodating mismatches, gaps, and wobble base pairings. It extends beyond the seed region to predict all possible miRNA target sites. We adjusted the threshold binding energy to −20 kcal/mol, set a score threshold of 100, and applied a gap-opening penalty (GOP) of −9 and a gap-extension penalty (GEP) of −4 [45].
StarMir: Uses miRNA binding data from CLIP studies in non-linear logistic prediction models. It excels at identifying seeded and unseeded target sites by considering thermodynamic, structural, and sequence features from SFold 2.2. The algorithm considers factors, such as the type of seed and site accessibility, and incorporates several parameters, including the Gibbs free energy change of the miRNA–mRNA target hybrid (ΔGhybrid) [43], the miRNA–mRNA target hybridisation (ΔGnucl), the total energy change of the hybridisation (ΔGtotal), and the LogitProb score [46,47].

### 4.3. Correlation-Based Assessments of miRNA Targets in the 3′ UTR

Using RNAhybrid “https://bibiserv.cebitec.uni-bielefeld.de/rnahybrid (accesed on 27 August 2024)”, 3′ UTR sequences were employed to predict the targets of host miRNAs from both humans and the mosquito species *Ae. aegypti* and *Cu. quinquefasciatus*. To assess which 3′ UTRs of flaviviruses exhibit a stronger affinity for host microRNAs, correlation analyses were performed based on the MFE hybridisation (kcal/mol). Spearman’s correlation coefficient was used to compare the interactions between miRNAs and the 3′ UTRs of the different flaviviruses.

Correlations approaching 1 indicated positive associations, suggesting that a lower MFE led to stronger interactions with specific viruses, suggesting shared miRNA binding sites among them. Conversely, correlations near −1 implied negative associations, indicating that a higher MFE resulted in weaker interactions between the miRNA and the correlated viruses, likely due to a lack of common binding sites. A correlation close to zero indicated an absence of a clear relationship between the MFE and miRNA–3′ UTR interactions.

### 4.4. Identification of miRNA Binding Sites

Following the initial data grouping, miRNAs that exhibited binding to the 3′ UTRs of all flaviviruses were subjected to further analyses. Typically, prevailing target prediction algorithms initiate a sequence search on 3′ UTRs, seeking regions with complementarity to miRNAs, ideally at their seed sites. However, this initial phase often results in thousands of potential target sites, accompanied by many false positives. To address this issue, most algorithms incorporate additional features such as MFE filters, the % mRNA–miRNA duplex complementarity, and evolutionary conservation to increase the specificity and to reduce false positives. Taking advantage of each algorithm, the candidates obtained from RNAhybrid were evaluated for their affinity for the 3′ UTRs of flaviviruses using three distinct algorithms: Inta-RNA “https://rna.informatik.uni-freiburg.de (accesed on 27 August 2024)”, miRanda “http://multimir.ucdenver.edu/ (accesed on 27 August 2024)”, and StarMir “https://sfold.wadsworth.org/cgi-bin/index.pl (accesed on 27 August 2024)”.

Positive candidates from the four algorithms were grouped, and miRNAs targeting all the 3′ UTRs of flaviviruses were selected as potential candidates. Finally, using MFE data information, we concatenated the information from each miRNA candidate to identify the optimal miRNA capable of binding the 3′ UTR of flavivirus genomes.

### 4.5. Computational Environments and Software

All data processing in this study was conducted in R and UNIX environments using specific packages. The miRNA network for the 3′ UTR of flavivirus genomes was created using Cytoscape 3.10.1 “https://cytoscape.org/ (accesed on 27 August 2024)”.

## 5. Conclusions

An analysis of human interactions revealed promising candidates, namely miR-6842 and miR-661, for the therapeutic targeting of flavivirus 3′ UTRs. In mosquito miRNA–flavivirus interactions, positive correlations suggest a strong affinity, whereas human interactions with various flaviviruses display negative correlations. Potential mosquito miRNA candidates, including miR-9-C, miR-2945-5p, miR-11924, miR-282-5p, and miR-79, warrant further exploration, offering the potential for viral transmission control strategies.

## Figures and Tables

**Table 1:** Position of human miRNA candidates in flavivirus genome 3′ UTRs.

miRNA	Flavivirus	Target Position	3′ UTR Target Sequence
miR-6842-5p	DENV1	10,508–10,536	actagtggttagaggagacccctcccaa
	DENV2	10,587–10,608	aggttagaggagacccccccga
	DENV3	10,571–10,590	aggttagaggagacccccc
	DENV4	10,513–10,535	aggttagaggagacccccccaa
	YFV	10,622–10,650	acctggtttctgggacctcccaccccag
	ZIKV	10,661–10,684	actagtggttagaggagacccccc
	JEV	10,831–10,852	aggttagaggagaccccgcat
	MVEV	10,862–10,886	aggttagaggagaccccactctca
	USUV	10,911–10,934	agaggttagaggagaccccgcat
	WNV1	10,807–10,824	aggttagaggagaccccg
	WNV2	10,807–10,824	aggttagaggagaccccg
miR-6791-5p	DENV1	10,428–10,455	gccgtgctgcctgtagctccatcgtgggga
	DENV2	10,344–10,365	cctgtgagccccgtccaagga
	DENV3	10,399–10,429	accgtgctgcctgtagctccgtcgtgggga
	DENV4	10,327–10,360	accgtgctgcctgtagctccgccaataatggga
	YFV	10,534–10,554	gttgtcagcccagaaccccac
	ZIKV	10,397–10,429	gtcaggcctgctagtcagccacagtttgggga
	JEV	10,655–10,679	gcggcctgcgcagccccaggagga
	MVEV	10,539–10,564	gctgcctgcgaccaaccccaggagg
	USUV	10,739–10,761	agcctgtacggccccaggagga
	WNV1	10,548–10,572	gctgcctgcgactcaaccccagga
	WNV2	10,483–10,510	gctgcctgcggctcaaccccaggagga
miR-6765-5p	DENV1	10,357–10,382	tatgctgcctgtgagccccgtccaa
	DENV2	10,336–10,362	ctatgctacctgtgagccccgtccaa
	DENV3	10,330–10,353	tgctgcctgtgagccccgtccaa
	DENV4	10,322–10,351	agcaaaccgtgctgcctgtagctccgcca
	YFV	10,436–10,467	ccacggctggagaaccgggctccgcacttaa
	ZIKV	10,745–10,771	cgctggccgccaggcacagatcgccg
	JEV	10,698–10,721	agcccccacggcccaagcctcgt
	MVEV	10,797–10,827	gagaccctgcggaagaaatgagtggcccaa
	USUV	10,766–10,797	ttaccaaagccgaaaggcccccacggcccaa
	WNV1	10,834–10,868	tgcacggcccagcctggctgaagctgtaggtcag
	WNV2	10,537–10,564	ccacgtaagccctcagaaccgtctcgg
miR-6762-3p	DENV1	10,551–10,578	cggggcccaacaccaggggaagctgta
	DENV2	10,539–10,566	atgggggcccaaggcgagatgaagctg
	DENV3	10,458–10,486	gtggggacgtaaaacctgggaggctgca
	DENV4	10,355–10,392	gggaggcgtaataatccccagggaggccatgcgccac
	YFV	10,719–10,753	aggagaccctccagggaacaaatagtgggaccat
	ZIKV	10,627–10,668	actggagactagctgtgaatctccagcagagggactagtgg
	JEV	10,760–10,800	aggttagaggagaccccgtggaaacaacaatatgcggccc
	MVEV	10,795–10,834	aggagaccctgcggaagaaatgagtggcccaagctcgcc
	USUV	10,844–10,874	aggagaccccgtggaacttaggtgcggccc
	WNV1	10,497–10,529	aggagaaagtcaggccgggaagttcccgccac
	WNV2	10,869–10,912	cctgggatagactaggggatcttctgctctgcacaaccagccac
miR-6756-5p	DENV1	10,424–10,450	aagccgtgctgcctgtagctccatcg
	DENV2	10,414–10,435	tgcagcctgtagctccacctg
	DENV3	10,325–10,354	aagctgtgctgcctgtgagccccgtccaa
	DENV4	10,328–10,350	cgtgctgcctgtagctccgcca
	YFV	10,663–10,686	gagcctccgctaccaccctccca
	ZIKV	10,590–10,613	aggtggcgaccttccccaccctt
	JEV	10,665–10,713	agccccaggaggactgggttaccaaagccgttgagcccccacggccca
	MVEV	10,701–10,749	aggccccaggaggactgggtaaacaaagccgtaaggcccccgcagcccg
	USUV	10,747–10,796	cggccccaggaggactgggttaccaaagccgaaaggcccccacggcccaa
	WNV1	10,752–10,775	cgccccacgcggccctagccccg
	WNV2	10,868–10,598	aggaccccacgtgctttagcctcaaagccca
miR-661	DENV1	10,518–10,563	aacgcagcagcggggcccaacaccaggggaagctgtaccctggtg
	DENV2	10,528–10,556	tcgcagcaacaatgggggcccaaggcga
	DENV3	10,514–10,535	aacgcagcagcggggcccgag
	DENV4	10,552–10,580	gacgctgggaaagaccagagatcctgct
	YFV	10,584–10,615	agtgcaggctgggacagccgacctccaggtt
	ZIKV	10,479–10,504	agtcaggccgagaacgccatggcac
	JEV	10,659–10,685	ctgcgcagccccaggaggactgggtt
	MVEV	10,743–10,780	agcccgggccgggaggaggtgatgcaaaccccggcga
	USUV	10,584–10,612	ggtgctgcctgcgactcaaccccaggcgg
	WNV1	10,915–10,945	agctgtaggtcaggggaaggactagaggtt
	WNV2	10,634–10,662	agtgcagtctgcgatagtgccccaggtg
miR-608	DENV1	10,549–10,583	agcggggcccaacaccaggggaagctgtaccctg
	DENV2	10,633–10,676	gggaaagaccagagatcctgctgtctcctcagcatcattcca
	DENV3	10,616–10,658	gggagagaccagagatcctgctgtctcctcagcatcattcca
	DENV4	10,558–10,600	gggaaagaccagagatcctgctgtctctgcaacatcaatcca
	YFV	10,659–10,683	aacggagcctccgctaccaccctc
	ZIKV	10,501–10,560	cacggaagaagccatgctgcctgtgagcccctcagaggacactgagtcaaaaaacccca
	JEV	10,495–10,522	gacggtgctgtctgcgtctcagtccca
	MVEV	10,532–10,558	gacggtgctgcctgcgaccaacccca
	USUV	10,582–10,609	gacggtgctgcctgcgactcaacccca
	WNV1	10,543–10,570	gacggtgctgcctgcgactcaacccca
	WNV2	10,478–10,505	gacggtgctgcctgcggctcaacccca
miR-4722-5p	DENV1	10,637–10,666	tgacgctgggagagaccagagatcctgct
	DENV2	10,626–10,656	tgacgctgggaaagaccagagatcctgctg
	DENV3	10,609–10,639	tgacgctgggagagaccagagatcctgctg
	DENV4	10,551–10,580	tgacgctgggaaagaccagagatcctgct
	YFV	10,587–10,611	gcaggctgggacagccgacctcca
	ZIKV	10,494–10,524	cgccatggcacggaagaagccatgctgcct
	JEV	10,616–10,657	gcggcctgcgcagccccaggaggactgggttaccaaagccg
	MVEV	10,726–10,750	aagccgtaaggcccccgcagcccg
	USUV	10,895–10,952	agaggttagaggagaccccgtggaacttaggtgcggcccaagccgtttccgaagctg
	WNV1	10,780–10,854	agaccccgcggtttaaagtgcacggcccagcctggct
	WNV2	10,865–10,896	cacctgggatagactaggggatcttctgctc

**Table 2:** Position of *Ae. aegypti* miRNA candidates in flavivirus genome 3′ UTRs.

miRNA	Flavivirus	Target Position	3′ UTR Flavivirus Sequence
miR-9c-5p	DENV1	10,635–10,660	attgacgctgggagagaccagagat
	DENV2	10,624–10,649	attgacgctgggaaagaccagagat
	DENV3	10,608–10,633	attgacgctgggagagaccagagat
	DENV4	10,550–10,575	attgacgctgggaaagaccagagat
	YFV	10,437–10,456	cacggctggagaaccgggc
	ZIKV	10,635–10,661	aacagcatattgacgctgggaaagac
miR-9b	DENV1	10,630–10,660	agcatattgacgctgggagagaccagagat
	DENV2	10,618–10,648	agcatattgacgctgggagagaccagagat
	DENV3	10,603–10,633	agcatattgacgctgggagagaccagagat
	DENV4	10,545–10,575	agcatattgacgctgggaaagaccagagat
	YFV	10,583–10,610	gcagtgcaggctgggacagccgacctc
	ZIKV	10,638–10,661	agcatattgacgctgggaaagac
miR-9a	DENV1	10,631–10,660	gcatattgacgctgggagagaccagagat
	DENV2	10,620–10,629	gcatattgacgctgggaaagaccagagat
	DENV3	10,604–10,633	gcatattgacgctgggagagaccagagat
	DENV4	10,546–10,566	gcatattgacgctgggaaagaccagagat
	YFV	10,437–10,456	cacggctggagaaccgggc
	ZIKV	10,635–10,667	aacagcatattgacgtgggaaagaccagagac
miR-92a-3p	DENV1	10,327–10,356	tcaggccggattaagccatagcacggtaa
	DENV2	10,542–10,571	ggggcccaaggcgagatgaagctgtagtc
	DENV3	10,422– 10,453	cgtggggacgtaaaacctgggaggctgcaaa
	DENV4	10,603–10,632	cacagagcgccgcaagatggattggtgtt
	YFV	10,587–10,621	tgcaggctgggacagccgacctccaggttgcgaa
	ZIKV	10,573–10,600	cgcaggatgggaaaagaaggtggcgac
miR-92b-3p	DENV1	10,326–10,350	tcaggccggattaagccatagcac
	DENV2	10,506–10,531	gcatggcgtagtggactagcggtta
	DENV3	10,421–10,452	cgtggggacgtaaaacctgggaggctgcaaa
	DENV4	10,539–10,566	gagcgccgcaagatggattggtgttgt
	YFV	10,587–10,621	tgcaggctgggacagccgacctccaggttgcgaa
	ZIKV	10,572–10,599	cgcaggatgggaaaagaaggtggcgac
miR-79-5p	DENV1	10,340–10,375	ccatagcacggtaagagctatgctgcctgtgagcc
	DENV2	10,528–10,528	cgcagcaacaatgggggcccaaggcg
	DENV3	10,516–10,537	cgcagcagcggggcccgagca
	DENV4	10,456–10,456	cgcagcaaaagggggcccgaagcc
	YFV	10,437–10,456	cacggctggagaaccgggc
	ZIKV	10,475–10,498	ctcatagtcaggccgagaacgcc
miR-316	DENV1	10,499–10,527	ggtagcagactagtggttagaggagacc
	DENV2	10,482–10,513	atggcgtagtggactagcggttagaggagac
	DENV3	10,477–10,502	agcagactagcggttagaggagacc
	DENV4	10,408–10,438	tggcatattggactagcggttagaggagac
	YFV	10,579–10,601	aggcagtgcaggctgggacag
	ZIKV	10,775–10,801	tcggcggccggtgtggggaaatccat
miR-2945-5p	DENV1	10,636–10,667	ttgacgctgggagagaccagagatcctgctg
	DENV2	10,625–10,656	ttgacgctgggaaagaccagagatcctgctg
	DENV3	10,609–10,640	ttgacgctgggaaagaccagagatcctgctg
	DENV4	10,551–10,582	ttgacgctgggaaagaccagagatcctgctg
	YFV	10,440–10,460	ggctggagaaccgggctccg
	ZIKV	10,466–10,497	ccatggcacggaagaagccatgctgcctgtg
miR-11924	DENV1	10,401–10,420	ggccgaaagccacggttcg
	DENV2	10,703–10,723	ctgttgaatcaacaggttct
	DENV3	10,687–10,707	ctgttgaatcaacaggttct
	DENV4	10,629–10,649	ttgttgatccaacaggttct
	YFV	10,599–10,616	agccgacctccaggttg
	ZIKV	10,752–10,788	cgccaggcacagatcgccgaacttcggcggccggtg
miR-11919-5p	DENV1	10,548–10,586	cagcggggcccaacaccaggggaagctgtaccctggtg
	DENV2	10,472–10,502	aagctgtacgcatggcgtagtggactagcg
	DENV3	10,459–10,488	agctgtacgcacggtgtagcagactagcg
	DENV4	10,600–10,624	aggcacagagcgccgcaagatgga
	YFV	10,580–10,632	aggcagtgcaggctgggacagccgacctccaggttgcgaaaaacctggttt
	ZIKV	10,428–10,465	aagctgtgcagcctgtaacccccccaggagaagctgg
miR-11-3p	DENV1	10,315–10,337	aggcaagaagtcaggccggatt
	DENV2	10,422–10,472	aggcacagaacgccagaaaatggaatggtgctgttgaatcaacaggttct
	DENV3	10,428–10,459	aggcacagaacgccagaaaatggaatggtgc
	DENV4	10,286–10,313	aggctattgaagtcaggccacttgtgc
	YFV	10,820–10,846	tcaagaataagcagacctttggatga
	ZIKV	10,620–10,647	gggcctgaactggagactagctgtgaa
miR-282-5p	DENV1	10,564–10,600	ccaggggaagctgtaccctggtggtaaggactagag
	DENV2	10,553–10,588	gagatgaagctgtagtctcgctggaaggactagag
	DENV3	10,426–10,449	ggacgtaaaacctgggaggctgc
	DENV4	10,457–10,492	aggaggaagctgtactcctggtggaaggactagag
	YFV	10,698–10,724	aagacggggtctagaggttagaggag
	ZIKV	10,423–10,468	tggggaaagctgtgcagcctgtaacccccccaggagaagctggga

**Table 3:** Position of *Cu. quinquefasciatus* miRNA candidates in flavivirus genome 3′ UTRs.

miRNA	Flavivirus	Target Position	3′ UTR Flavivirus Sequence
miR-993-5p	JEV	10,469–10,496	caaaagctgccaccggatactgggtag
	MVEV	10,699–10,723	tgaggccccaggaggactgggtaa
	USUV	10,748–10,769	cggccccaggaggactgggtt
	WNV1	10,844–10,873	cagcctggctgaagctgtaggtcagggg
	WNV2	10,504–10,650	aggaggactgggtgaccaaagctgcgaggtga
miR-989	JEV	10,498–10,523	ggtgctgtctgcgtctcagtcccag
	MVEV	10,651–10,670	agcccgtgtcagatcgcga
	USUV	10,709–10,731	ggccgcaaagcgccacttcgcc
	WNV1	10,973–10,994	agccacacggcacagtgcgcc
	WNV2	10,906–10,927	agccacacggcacagtgcgcc
miR-79	JEV	10,914–10,945	acatcagctactaggcacagagcgccgaagt
	MVEV	10,653–10,692	ccgtgtcagatcgcgaaagcgccacttcgccgaggagtg
	USUV	10,865–10,892	ggtgcggcccaagccgtttccgaagct
	WNV1	10,975–11,007	ccacacggcacagtgcgccgacaatggtggct
	WNV2	10,784–10,807	tggctgaagctgtaagccaaggg
miR-71-3p	JEV	10,818–10,846	ggtggaaggactagaggttagaggagac
	MVEV	10,778–10,803	cgaaggactagaggttagaggagac
	USUV	10,553–10,582	tcgtggaaggactagaggttagaggagac
	WNV1	10,870–10,895	ggaaggactagaggttagtggagac
	WNV2	10,805–10,830	ggaaggactagaggttagaggagac
miR-305-5p	JEV	10,930–10,963	cagagcgccgaagtatgtagctggtggtgagga
	MVEV	10,969–11,002	cgagcgccgaacactgtgactgatgggggagaa
	USUV	10,553–10,582	cagggcaacctgccaccggaagttgagta
	WNV1	10,985–11,018	cagtgcgccgacaatggtggctggtggtgcgag
	WNV2	10,618–10,644	tgtgccactctgcggagagtgcagtc
miR-285-5p	JEV	10,591–10,609	cctgctcactggaagttg
	MVEV	10,513–10,553	gctgccaccgaaggttggtagacggtg
	USUV	10,561–10,582	cctgccaccggaagttgagta
	WNV1	10,956–10,979	ttctgctctgcacaaccagccac
	WNV2	10,889–10,912	ttctgctctgcacaaccagccac
miR-283	JEV	10,669–10,702	ccccaggaggactgggttaccaaagccgttgag
	MVEV	10,554–10,597	ccccaggaggactgggttaccaaagctgattctccacggttgg
	USUV	10,603–10,646	ccccaggcggactgggttaacaaagctgaccgctgatgatgg
	WNV1	10,565–10,585	ccccaggaggactgggtgaa
	WNV2	10,500–10,520	ccccaggaggactgggtgac
miR-278	JEV	10,578–10,597	tcggaagtaggtccctgct
	MVEV	10,616–10,637	tcggaagaggagtccctgcca
	USUV	10,607–10,641	caggcggactgggttaacaaagctgaccgctgat
	WNV1	10,498–10,517	aggagaaagtcaggccggg
	WNV2	10,560–10,579	tcggaaggaggaccccacg
miR-210-3p	JEV	10,693–10,720	aagccgttgagcccccacggcccaagc
	MVEV	10,649–10,673	aagcccgtgtcagatcgcgaaagc
	USUV	10,697–10,718	cagcccgtgtcaggccgcaaa
	WNV1	10,657–10,684	aagcccaatgtcagaccacgctacggc
	WNV2	10,579–10,600	aagcccagtgtcagaccacac
miR-2	JEV	10,936–10,962	cgccgaagtatgtagctggtggtgag
	MVEV	10,827–10,855	agctcgccgaagctgtaaggcgggtgga
	USUV	10,623–10,646	acaaagctgaccgctgatgatgg
	WNV1	10,990–11,015	cgccgacaatggtggctggtggtgc
	WNV2	10,923–10,948	cgccgacataggtggctggtggtgc
miR-14	JEV	10,809–10,834	tgtagaggaggtggaaggactagag
	MVEV	10,684–10,720	gaggagtgcaatctgtgaggccccaggaggactggg
	USUV	10,731–10,767	aaggagtgcagcctgtacggccccaggaggactggg
	WNV1	10,859–10,883	tgtaggtcaggggaaggactagag
	WNV2	10,726–10,757	agggagaagggactagaggttagaggagacc
miR-1175-5p	JEV	10,886–10,911	agactgggagatcttctgctctatc
	MVEV	10,923–10,949	agactaggagatcttctgctctattc
	USUV	10,975–11,001	agactaggagatcttctgctctattc
	WNV1	10,943–10,968	agactaggagatcttctgctctgca
	WNV2	10,876–10,901	agactaggggatcttctgctctgca

**Table 4:** Functions of miRNAs detected in mosquitoes that interact with 3′ UTR of flaviviruses.

miRNA	Organism	Function	Reference
miR-9c	*Drosophila melanogaster*	Highly expressed in brain. Participates in memory reduction.	[58]
	*Drosophila melanogaster*	Highly expressed in eggs. Participates in transcript clearance in the maternal-to-zygotic transition process during development.	[59]
	*Drosophila melanogaster*	Induction of cellular proliferation by inhibition of PntP1, an inductor of Dap.	[60]
	*Scylla paramamosain*	Negative regulation of the ERK pathway that is important in ovarian development.	[61]
	*Scylla paramamosain*	Regulation of the cell cycle in ovarian development by inhibition of cyclin A and CDK1.	[68]
miR-282-5p	*Bombyx mori*	Inhibition of chitinase 5 during the moulting process.	[69]
miR-79	*Ixodes scapularis*	Enhances *Anaplasma phagocytophilum* infection in ISE6 cells through the inhibition of Roundabout protein 2.	[74]
	*Drosophila melanogaster*	Suppresses tumour growth through activation of the JNK signaling pathway by inhibition of the RNF146 protein.	[71]
	*Drosophila melanogaster*	Induction of cellular proliferation by inhibition of PntP1, an inductor of Dap.	[60]
	*Bombyx mori*	Inhibits *BmEm4*, a gene involved in development and metamorphosis.	[72]
	*Apostichopus japonicus*	Participates in metabolic rate suppression during aestivation.	[73]
	*Caenorhabditis elegans*	Controls the hermaphrodite-specific neuron migration during embryogenesis by targeting SQV-5 and SQV-7, enzymes involved in glycosaminoglycan biosynthesis	[70]

**Table 5:** Functions of miRNAs detected in humans that interact with the 3′ UTR of flaviviruses.

miRNA	Function	Reference
miR-6842-5p	Reduction of proliferation, migration, and the formation of capillary-like structures in HUVEC cells by suppression of AKT2.	[77]
miR-661	Upregulated in squamous cell carcinoma.	[85]
	Upregulated in non-small-cell lung cancer and promotes proliferation and invasion through the RUNX3 and RB1/E2F pathways but inhibits apoptosis through DOK7. Biomarker for diagnosis and prognosis.	[86,87,88]
	Promotes migration and proliferation of lung cancer cells by targeting ADRA1A.	[89]
	Binds to Tumour suppressor candidate-2 pseudogene (TSC2P) in esophageal squamous cell carcinoma.	[90]
	Downregulated in cervical carcinoma tissues.	[91]
	Antitumoural effect by promoting apoptosis in osteosarcoma cells through binding to Cytochrome C1.	[92]
	Downregulated in breast cancer tissues and a breast epithelial cell line. Inhibits proliferation and glycolysis by targeting HMGA1, regulates the expression of metastatic tumour antigen 1, and has antitumoural effect by binding to Mdm2 and Mdm4, thus stabilising p53. However, it is upregulated in breast cancer tissues from patients with metastasis and in triple-negative breast cancer from LatinAmerican patients.	[93,94,95,96,97]
	Downregulated in HUVEC cells stimulated with extracellular vesicles from anti-PR3-activated neutrophils (granulomatosis with polyangiitis).	[98]
	Upregulated in patients with myelodysplastic syndrome. It induces apoptosis through the p53 pathway.	[99]
	Inhibits proliferation and migration of vascular smooth muscle cells by targeting SYK mRNA.	[100]
	Downregulated in macrophages from patients with varicose veins.	[101]
	Downregulated in glioma tissues. Inhibits metastasis and promotes apoptosis in glioma cells by targeting RAB3D, and it degrades *HOXD-AS2*, a lncRNA that promotes cell proliferation and cell cycle progression.	[102,103,104]
	Upregulated in pancreatic ductal adenocarcinoma and associated with a bad prognosis but downregulated in patients with other pancreatic cancers. It promotes cell proliferation through activation of the Wnt signalling pathway.	[105,106]
	Downregulated in sera from patients with Alzheimer’s disease.	[107]
	Overexpressed in patients with hepatocellular carcinoma. It inhibits PTPN11, a tumour suppressive tyrosine phosphatase.	[108,109]
	Present in high levels in blood samples of large-for-gestational age mothers during the second trimester of pregnancy.	[110]
	Overexpressed in non-implanted blastocysts. It inhibits embryo–endometrial adhesion by downregulation of poliovirus receptor-related 1.	[111]
	Overexpressed in the blastocoel fluid of aneuploid embryos.	[112]
	Overexpressed in serum from patients with incomplete Sjögren’s syndrome.	[113]
	Downregulated in human keloid tissues. It inhibits expression of FGF2, a factor involved in keloid progression.	[114]
	Increased in patients with diabetes mellitus type 2 with microvascular complications or foot ulcers. Putative biomarker.	[115,116]
	Upregulated in ovarian cancer tissues acting as a tumour promoter through the NPP5J-induced AKT pathway.	[117]

## Data Availability

Data generated or analysed during this study are included in this article. Further inquiries can be directed to the corresponding authors.

## Supplementary Materials

The following supporting information can be downloaded at: https://www.mdpi.com/article/10.3390/ijms251810135/s1.
